# Anti-Inflammatory Effects of Epoxyeicosatrienoic Acids

**DOI:** 10.1155/2012/605101

**Published:** 2012-07-16

**Authors:** Scott J. Thomson, Ara Askari, David Bishop-Bailey

**Affiliations:** Translational Medicine and Therapeutics, William Harvey Research Institute, Barts and The London School of Medicine and Dentistry, Queen Mary University of London, Charterhouse Square, London EC1M 6BQ, UK

## Abstract

Epoxyeicosatrienoic acids (EETs) are generated by the activity of both selective and also more general cytochrome p450 (CYP) enzymes on arachidonic acid and inactivated largely by soluble epoxide hydrolase (sEH), which converts them to their corresponding dihydroxyeicosatrienoic acids (DHETs). EETs have been shown to have a diverse range of effects on the vasculature including relaxation of vascular tone, cellular proliferation, and angiogenesis as well as the migration of smooth muscle cells. This paper will highlight the growing evidence that EETs also mediate a number of anti-inflammatory effects in the cardiovascular system. In particular, numerous studies have demonstrated that potentiation of EET activity using different methods can inhibit inflammatory gene expression and signalling pathways in endothelial cells and monocytes and in models of cardiovascular diseases. The mechanisms by which EETs mediate their effects are largely unknown but may include direct binding to peroxisome proliferator-activated receptors (PPARs), G-protein coupled receptors (GPCRs), or transient receptor potential (TRP) channels, which initiate anti-inflammatory signalling cascades.

## 1. Introduction

Cardiovascular diseases such as atherosclerosis have a strong inflammatory component. Inflammation in the vascular wall may be initiated by endothelial dysfunction and the accumulation of toxic oxidised circulating lipids [1]. Inflammatory mediators such as TNF*α* and IL-1*β* secreted, which induces the upregulation of cell adhesion molecules, facilitates leukocyte recruitment in to the vascular wall [2, 3] and stimulates vascular smooth muscle cell migration and proliferation [4]. Circulating monocytes not only respond to inflammatory stimuli by producing large amounts of inflammatory mediators but they are also crucial for effective activation of lymphocytes and adaptive immunity. The hallmark of advanced unstable atherosclerotic lesions is that they are monocyte/macrophage rich and highly inflammatory.

Inflammatory responses are normally promptly terminated since excessive or prolonged inflammation can lead to chronic pathological conditions such as cardiovascular diseases, Crohn's disease, rheumatoid arthritis, or cancer. Although there have been many new treatments recently developed to combat inflammatory diseases, some of these treatments are either very expensive and/or not effective in subsets of patients. Therefore, it is important to continue to investigate mechanisms that regulate inflammatory responses as they may open up novel therapeutic targets. There is a growing list of evidence that the epoxygenase pathway of arachidonic acid metabolism, which generates epoxyeicosatrienoic acids (EETs), exerts anti-inflammatory effects that may be harnessed to treat disease. This paper will summarise that evidence and highlight outstanding questions that remain to be answered.

## 2. Overview of the Epoxygenase Pathway of Arachidonic Acid Metabolism

Arachidonic acid is an omega-6 polyunsaturated long chain fatty acid that contains 20 carbon atoms and four *cis*-double bonds and possesses a carboxyl group and a methyl group at respective ends of the molecule. The double bonds are located between carbons 5-6, 8-9, 11-12, and 14-15 relative to the carboxyl group. Therefore, its chemical name is *all-cis*-5,8,11,14-eicosatetraenoic acid and its lipid name is 20 : 4 (n-6). The *cis*-configuration of the four double bonds causes the arachidonic acid backbone to significantly bend. In contrast, double bonds in the *trans*-configuration or saturated arachidonic acid result in structurally unbent or flexible backbones.

Experiments performed more than 30 years ago showed that incubations of radio-labelled arachidonic acid with microsomal preparations derived from a variety of tissues including liver [5, 6], kidney [7], hypothalamus [8], and anterior pituitary [9] resulted in the formation of EETs. This “epoxygenase” reaction required cytochrome p450 (CYP) enzymes and utilised NADPH and oxygen in a 1 : 1 stoichiometric ratio [5]. One atom of molecular oxygen is incorporated into one of the four double bonds of arachidonic acid retaining the *cis*-geometry and yielding four potential EETs, that is, 5,6-EET, 8,9-EET, 11,12-EET, or 14,15-EET, respectively. Furthermore, each EET can be present in either the S/R or R/S stereoconfiguration, thus eight potential EETs can be formed.

### 2.1. Epoxygenation of Arachidonic Acid Performed by Specific CYPs

CYP enzymes catalyze the oxidation of organic substances, as well as xenobiotics. Altogether, 57 putative CYP genes have been identified in man (by comparison mice have 103 and rat 89 CYP, resp.) that are divided into 15 subfamilies [10]. Attempts have been made to classify human CYP genes by substrate; however, a more systematic nomenclature is generally used since the true physiological roles of many of these genes are still unknown [11]. To date, at least 12 human CYP genes have been reported to possess epoxygenase activity, although most studies have been focussed on the CYP2C and CYP2J families, which are considered the major epoxygenase enzymes.

### 2.2. CYP2C

One of the earliest studies using recombinant human CYP compared the metabolic profiles of the CYP2C8 and CYP2C9 enzymes [12], which are 77% identical at the amino acid level. Despite their high similarity, CYP2C8 and CYP2C9 exhibit both regio- and stereoselective differences in their epoxygenation of arachidonic acid. For instance, CYP2C8 produced only 14,15-EET and 11,12-EET at a 1.25 : 1 ratio, which represented 68% of the total metabolites measured. By contrast, CYP2C9 produced 14,15-EET 11,12-EET and 8,9-EET at a ratio of 2.3 : 1 : 0.5, which represented 69% of total metabolites. Furthermore, with respect to stereoselectivity, CYP2C8 was 81% selective for the 11(R),12(S)-EET configuration, whereas CYP2C9 was 70% selective for the 11(S),12(R)-EET configuration [12]. These CYP enzymes also carry out other reactions including allylic hydroxylation on arachidonic acid and other fatty acids.

### 2.3. CYP2J2

Epoxygenase activity of human CYP2J2 was first demonstrated by the Zeldin lab, who initially cloned and characterised the gene [13]. Recombinant CYP2J2 metabolised arachidonic acid to all four potential epoxygenase products, with 14,15-EET being the predominant metabolite formed. CYP2J2 was found to be highly expressed in the heart, and EETs were produced in similar proportions as recombinant CYP2J2 suggesting that CYP2J2 played a major role in EET generation in the heart *in vivo* [13]. CYP2J2 expression is also seen in kidney, liver and muscle tissues [13], and, to a lesser extent, in the gut [14].

### 2.4. Other CYPs

A comprehensive comparison study by Rifkind and colleagues examined the epoxygenase activity of a panel of 10 CYP proteins by overexpressing them in HepG2 cells and measuring metabolic products. CYP 2C8, 2C9, 1A2, and 2E1 principally produced epoxygenase products. By contrast, CYP2D6 was inactive, while CYPs 2A6, 3A3, 3A4, and 3A5 had minimal epoxygenase activity [15]. CYP3A4 has also been shown to make the epoxygenase products 8,9-EET, 11,12-EET, and 14,15-EET, respectively, in several breast cancer cell lines [16]. Other CYPs that have been shown to possess epoxygenase activity include CYP1A, CYP2B1 and CYP2B2 [17] and CYP2B12 [18], CYP2C8, CYP2C9, CYP2D18 [19], CYP2N1 and CYP2N2 [20], and rat CYP4A2 and 4A3 [21]. The full extent of the epoxygenase activity of these enzymes and the physiological consequences of any activity is, however, poorly understood.

## 3. Soluble Epoxide Hydrolase 

Once formed, EETs are unstable because they are rapidly metabolised. The main catabolic pathway is the conversion of EETs into dihydroxyeicosatrienoic acid (DHETs), catalysed by soluble epoxide hydrolase (sEH) [22]. DHETs are generally considered to be less active; however, they have been shown to exert vasodilatory effects on coronary arteries [23]. DHETS are far more polar than their corresponding EETs and quickly diffuse out of tissues as the 1, diols or conjugates of them. Other pathways of EET metabolism include chain elongation, *β*-oxidation, and *ω*-oxidation [24]. 5,6 EET and 8,9 EET are substrates for COX enzymes [25] and can also be incorporated into membrane phospholipids. DHETs have a lower binding affinity for phospholipids which may account for its relatively increased plasma levels [26].

Recently, the damaging cardiovascular risk factor homocysteine has been shown to upregulate sEH in endothelial cells and promote a proinflammatory environment [27]. In contrast, elevating the levels of endogenous CYP products by removing (sEH knockout mice) or inhibiting soluble epoxide hydrolase (sEH-1) has been shown to reduce neointima formation [28], atherosclerosis and abdominal aortic aneurysm development, dyslipidaemia in hyperlipidaemic mice [29], and reduce hypertension [30] and diabetes [31] in different mouse models. A number of sEH inhibitors have now been developed and are moving towards clinical trials for a variety of disorders related to cardiovascular disease.

## 4. Epoxygenases in Vascular and Inflammatory Cells

CYP2C mediated generation of 11,12 EET has also been documented in porcine coronary arteries [32], and CYP2C enzymes have been found expressed in endothelial cells [33], and in primary human monocytes and M1 (CYP2C8) and M2 macrophages (CYP2C8 and CYP2C9) [34].

CYP2J2 immunoreactivity is seen in the endothelial and smooth muscle cell layers of human coronary arteries [35], as well as in the human monocytic cell lines THP-1 and U937, primary monocytes and M1 and M2 macrophages [34], and the endothelial cell line EA hy.926s [36]. Interestingly, neither CYP2J2 nor CYP2C8 mRNA expression was detected in human polymorphonuclear cells [34]. More recently, the increased risk of coronary artery disease was shown to be associated with a polymorphism in the promoter of *CYP2J2* gene in some populations, which decreases the expression of the enzyme [37].

## 5. Epoxygenases and EETs Suppress**** Inflammation

EETs have been shown to exert multiple biological effects on the vasculature including proproliferation and angiogenic effects [38]. EETs have also been hypothesized as endothelium-derived hyperpolarizing factors, as they hyperpolarize and relax vascular smooth muscle cells by activating calcium-activated potassium channels [32]. However, a number of the anti-inflammatory activities of EETs on inflammatory cells, as discussed below, appear independent of any cellular hyperpolarisation [35].

### 5.1. Endothelial Cells

Overexpression of CYP2J2 in human and bovine endothelial cells inhibits TNF*α*-induced VCAM-1 [39] and VCAM-1 promoter activity in reporter assays [35]. Treatment with the epoxygenase inhibitor SKF525A reversed the effects of CYP2J2 overexpression on VCAM-1 promoter activity [35]. Exogenous EETs also exert the same effects as CYP2J2 overexpression, although different EETs can have different selectivities. In human endothelial cells, 11,12-EET significantly inhibited VCAM-1 expression in response to TNF*α*, IL-1*α*, or LPS. By contrast, 14,15-EET had negligible effect, while 5,6-EET, 8,9-EET, and 11,12-DHET all inhibited to varying degrees but to a lesser extent than 11,12-EET. 11,12-EET also inhibited TNF*α*-induced E-selectin and ICAM-1 expression [35]. Mice engineered to overexpress the human epoxygenase genes *CYP2J2* or *CYP2C8,* respectively, were generated to investigate their roles in endothelial cells. Primary pulmonary endothelial cells derived from these mice showed reduced levels of LPS-induced adhesion molecule and chemokine gene expression. Furthermore, these anti-inflammatory effects were inhibited by treatment with the epoxygenase inhibitor MS-PPOH and a putative EET receptor antagonist 14,15-EEZE [40].

### 5.2. Monocytic Cells

Similar to endothelial cells, EET activity has also been shown to antagonise inflammatory signals in monocytic cells. Phorbol ester treatment of THP-1 led to a 4-fold increase in CYP2J2 expression between 3–7 days after stimulation, suggesting that endogenous expression of CYP2J2 may regulate inflammatory responses in these cells [36]. Addition of 8,9-EET or 11,12-EET inhibited basal TNF secretion from THP1 cells by about 90% and 40%, respectively [34]. Similarly, the epoxygenase inhibitor SKF525A led to a concentration-dependent superinduction of LPS-induced PGE_2_ in rat monocytes and COX-2 in mouse and human monocytes [41]. Consistent with these findings, exogenous 11,12-EET dose dependently inhibited LPS-induced PGE_2_ and attenuated SKF-mediated superinduction. 11,12-EET also inhibited LPS-induced COX-2 activity and expression [41]. EETs can, therefore, both compete with arachidonic acid for the binding site in COX enzymes as well as inhibit the inflammation induced induction of COX-2 expression. A study found that EETs were detected in human peritoneal macrophages under basal conditions, but not following zymosan treatment, which caused a shift to prostaglandin synthesis [42].

### 5.3. Leukocyte Endothelial Cell Interactions

Several studies have demonstrated that EETs can regulate functional interaction between leukocytes and endothelial cells. Treatment of endothelial cells with 14,15-EET significantly enhanced attachment of the monocytic cell line U937 [43]. Pretreatment of endothelial cells with EETs alone or in combination with PMA had negligible effects on adherence of PMNs. However, cotreatment of EETs and PMA led to a concentration-dependent decrease in adherence of PMNs when cocultured with endothelial cells [44]. 11,12-EET, but not 14,15-EET, was shown to inhibit adherence of monocytic cells in an *ex vivo* model. Mice were treated with TNF*α* alone or in combination with either 11,12-EET or 14,15-EET, and carotid arteries were removed and incubated with U937 cells. The level of inhibition of adherent cells was comparable to that of treatment with a blocking VCAM-1 antibody [35]. PBMCs derived from mice systemically overexpressing human CYP2J2 via *in vivo* gene delivery were significantly less adherent to TNF*α*-treated HUVECs compared to control PBMCs [39].

### 5.4. *In Vivo* Models

There have been conflicting reports on the effects of EETs in acute models of inflammation *in vivo*. Rats injected with TNF*α* showed elevated plasma levels of adhesion molecules and inflammatory cytokines, and decreased levels of the anti-inflammatory mediator IL-10. However, these effects were significantly reduced by systemic overexpression of human CYP2J2 [39], suggesting that EETs act as anti-inflammatory mediators. Similarly, TNF*α*-treated human bronchi also showed reduced inflammation when treated with 14,15-EET [45]. LPS responses of wild-type mice have also been compared to sEH−/− null mice or mice that had endothelial-specific overexpression of the human CYP2J2 or CYP2C8. All three genetically modified mice had reduced levels of inflammatory gene expression and neutrophil recruitment in the lung following LPS injection. Moreover, these effects correlated with decreased activation of the key transcription factor NF-*κ*B [40]. By contrast, another study found that to sEH−/− null mice were not protected from LPS-induced inflammatory gene expression or neutrophil recruitment in the liver, and that treatment with the sEH inhibitor AUDA also had minimal effect liver inflammation, despite higher levels of endogenous EETs [46]. This suggests that either the effects of EETs are organ specific, or that liver is more susceptible to endotoxin shock.

## 6. Mechanisms of EET Action

### 6.1. NF-*κ*B Inhibition

The mechanisms by which EETs mediate their anti-inflammatory effects remain ill-defined, but there are several reports that they can inhibit activation of NF-*κ*B, a key transcription factor for inflammatory gene induction. In mammals NF-*κ*B comprises five subunits, with the RelA (p65) subunit being expressed in most cell types. Under basal conditions, NF-*κ*B dimers are localised in the cytoplasm due to interactions with I*κ*B (inhibitor of NF-*κ*B) proteins. Signalling cascades induced by inflammatory descend on the IKK (inhibitor of NF-*κ*B kinase) complex, which phosphorylates I*κ*B. This tags I*κ*B for subsequent ubiquitination and degradation by the proteosome, which, in turn, facilitates NF-*κ*B nuclear translocation where it binds to its cognate binding elements to activate transcription [47, 48].

11,12-EET inhibits NF-*κ*B reporter activity in both HEK293 cells [34] and human endothelial cells [35] following stimulation. Furthermore, 11,12-EET also inhibited TNF*α*-induced RelA nuclear translocation, I*κ*B*α* degradation, and IKK*α* activity, respectively [35], indicating that EET-mediated inhibition of NF-*κ*B occurs upstream of IKK. Interestingly, 14,15-EET was also shown to inhibit the TNF*α*-induced degradation of I*κ*B*α* in primary human lung tissue [45] but had no effect on NF-*κ*B reporter activity in HEK293s, suggesting that 14,15-EET may act in a cell type-specific manner. Similarly, 8,9-EET and 11,12-EET inhibited NF-*κ*B reporter gene activity in HEK293 cells [34]. In contrast to CYP2J2, CYP2C9 increased NF-*κ*B activity in human vascular endothelium via superoxide generation, potentially giving this CYP a proinflammatory profile [49].

### 6.2. STAT3

EETs can also activate STAT3 in human breast cancer cell lines, with 14,15-EET promoting STAT3 tyrosine-705 phosphorylation and nuclear translocation [16]. Activation of STAT3 was shown to be dependent on cell proliferation, which led the authors to conclude that 14,15-EET may be involved in an autocrine/paracrine pathway driving cell growth. Interestingly, the anti-inflammatory effects of IL-10 in macrophages are also dependent on STAT3 tyrosine-705 phosphorylation [50]. Taken together, these results suggest that the anti-inflammatory effects of EETs may be mediated by activation of STAT3, in addition to the inhibition of NF-*κ*B.

### 6.3. EETs as PPAR Agonists

PPARs are a subfamily of the nuclear receptor superfamily that comprises three ligand-activated transcription factors: PPAR*α* (NR1C1), PPAR*β*/*δ* (NR1C2), and PPAR*γ* (NR1C3). Upon ligand binding, they form heterodimers with the retinoid X receptor and bind to specific response elements in gene promoters to upregulate gene transcription [51]. PPARs have been shown to regulate diverse physiological processes such as fatty acid and glucose metabolism, angiogenesis, and cellular proliferation and differentiation, in addition to inflammation. PPAR ligands include a variety of fatty acids, and there has been recent evidence that metabolites of the epoxygenase pathway can activate PPAR receptors.

The omega-alcohol of 14,15-EET, 20,14,15-HEET, or a 1 : 4 mixture of the omega-alcohols of 8,9- and 11,12-EETs activated human and mouse PPAR*α* in transient transfection assays, suggesting a role for them as endogenous ligands for these orphan nuclear receptors [52]. Overexpression of human CYP2J2 in HEK293 cells resulted in a synergistic activation of PPAR*α*, -*β*/*δ* and, -*γ* reporter gene activity. 8,9-EET and 11,12-EET, but not 14,15-EET, (in contrast to its hydroxy metabolite 20,14,15-HEET) were able to induce PPAR*α* reporter activity [53]. Furthermore, IL-1*β*-induced NF-*κ*B reporter activity and COX-2 mRNA induction in HEK293 cells was significantly inhibited cells expressing of CYP2J2 and PPAR*α*.

Competition and direct binding assays subsequently revealed that EETs bind to the ligand-binding domain of PPAR*γ* with K(d) in the *μ*M range. In the presence of the sEH inhibitor AUDA, EETs increased PPAR*γ* transcription activity in endothelial cells and 3T3-L1 preadipocytes. In endothelial cells, AUDA enhanced, but overexpression of sEH reduced laminar flow-induced PPAR*γ* activity, EET generation, and the inhibition of VCAM-1 expression [54]. PPARs, therefore, represent a viable receptor target for the anti-inflammatory effects of EETs. However, it should be noted that AUDA may exert multiple effects in addition to sEH inhibition. It has been shown to act both as a PPAR agonist [55] and a EET mimetic [56]; therefore, results using AUDA should be cautiously interpreted.

### 6.4. GPCRs

For some time it has been suggested that EETs might mediate many of their effects via binding to a putative G-protein coupled receptor(s) (GPCRs). For example, 11,12-EET produced a 0.5- to 10-fold increase in the activity of the KCa channels in smooth muscle cells derived from bovine coronary arteries, which was dependent on the presence of GTP [57]. Furthermore, blocking antibodies against GS*α*, but not G*βγ* or anti-Gi*α*, were able to inhibit the activation induced by 11,12-EET [57]. Using radio-ligand binding, 14,15-EET has been shown to have a high affinity for a receptor expressed on guinea pig-derived mononuclear cells, which was purported to be a G-protein coupled receptor that stimulated cAMP production [58]. This putative GPCR-cAMP pathway remains elusive but may represent a novel anti-inflammatory pathway by which EETs act.

### 6.5. TRPV1 and EETs

TRPV4 is a cation channel of the “transient receptor potential” (TRP) family that functions as a Ca^2+^ entry channel, that is expressed in smooth muscle cells, endothelial cells, as well as in perivascular nerves. CYP-dependent generation of 5,6-EET can activate TRPV4 in murine endothelial cells and is a possible contributing mechanism to the hyperpolarising effects of EETs [59]. Additionally, 11,12-EET can activate TRPV4 channels in smooth muscle cells from rat cerebral arteries [60], and 5,6-EET and 8,9-EET can activate TRPV4 in human endothelial cells [61]. Although activated by EETs, there is little evidence that activation of TRPV4 is anti-inflammatory, though it does lead to vasodilation via nitric oxide, prostacyclin, and intermediate/small conductance K+ channel-dependent pathways, and in vascular smooth muscle, large conductance K+ channel activation, and hyperpolarization [62].

## 7. Summary and Outlook

More than 100 metabolites derived from arachidonic acid have been described, with the best characterised coming from the COX and LOX pathways which generate prostanoids and leukotrienes, respectively [63]. Knowledge of these pathways has led to several important therapeutic breakthroughs such as COX inhibitors which are used to treat pain and inflammation and leukotriene antagonists that have been used to treat asthma. By contrast, much less is known about the epoxygenase pathway of arachidonic acid metabolism, although as outlined in this paper, EETs can exert a number of cardio-protective anti-inflammatory effects on vascular cells such as endothelial cells and monocytes. These include inhibition of proinflammatory mediators and cell adhesion molecules. Indeed, a recent study has measured epoxygenase products in atherosclerotic patients [64]. Compared to healthy volunteers, both obese and nonobese CAD patients had significantly higher plasma EETs [64], suggesting that this is a compensation mechanism to protect against ongoing vascular inflammation.

Although elevating epoxygenase products via sEH inhibition have been shown to be beneficial in a wide variety of animal models of cardiovascular disease, the mechanisms through which these effects are mediated are still largely unknown, although NF-*κ*B and STAT3 have both been implicated. However, several fundamental question regarding the role of EETs in vascular inflammation remain unanswered. Firstly, it is clear that CYP epoxygenases can act on substrates other than arachidonic acid, such as cardio-protective fish oils. Eicosapentaenoic acid for example is an omega-3 long chain fatty acid that differs from arachidonic acid by the addition of one extra double bond at the 17-18 carbon position. Epoxygenation of eicosapentaenoic acid by CYP enzymes generates 17,18-epoxyeicosatrienoic acid, which has a hyperpolarising effect on bronchial smooth muscle cells *in vitro *and* in vivo *[65]. Similarly, linoleic acid, which is the major dietary fat, can be epoxygenated by CYP enzymes resulting in potent metabolites which are probably proinflammatory in nature. However, little is known regarding the function of many of these alternative “epoxygenase” metabolites have on the cardiovascular system during inflammation. Secondly, the full range of epoxygenase activity by CYP enzymes in healthy and diseased physiological settings is still not completely understood and remains a significant barrier to progress in the field. Thirdly, and probably most importantly, definitive identification of a specific receptor that mediates the activities of EETs is essential to fully understand the epoxygenase pathway, and will help to elucidate new therapies for cardiovascular diseases in the future.
